# Supplementary material for: AtlasXplore: a web platform for visualizing and sharing spatial epigenome data
Source: Bioinformatics. 2023 Jul 21;39(8):btad447. doi: 10.1093/bioinformatics/btad447 (PMC10394123; doi:10.1093/bioinformatics/btad447)
Supplement: btad447_Supplementary_Data [file btad447_supplementary_data.docx]

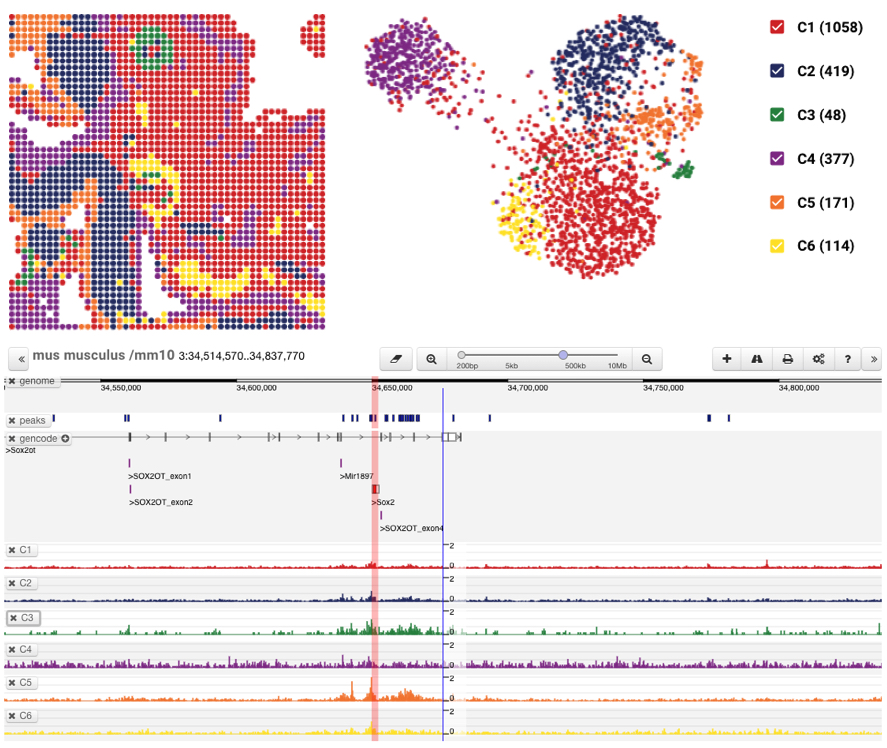


**Figure S1**. Spatial and Umap plots (top) and genome browser view of Sox2 (bottom) for the spatial ATAC mouse embryo data.
